# Abnormal costimulatory phenotype and function of dendritic cells before and after the onset of severe murine lupus

**DOI:** 10.1186/ar1911

**Published:** 2006-02-28

**Authors:** Lucrezia Colonna, Joudy-Ann Dinnall, Debra K Shivers, Lorenza Frisoni, Roberto Caricchio, Stefania Gallucci

**Affiliations:** 1 Laboratory of Dendritic Cell Biology, Division of Rheumatology, Joseph Stokes' Jr. Research Institute, Children's Hospital of Philadelphia, 3615 Civic Center Boulevard, Philadelphia, PA 19104-4318, USA; 2 Division of Rheumatology, School of Medicine, University of Pennsylvania, 751 BRB II/III, 421 Curie Blvd, Philadelphia, PA 19104, USA

## Abstract

We analyzed the activation and function of dendritic cells (DCs) in the spleens of diseased, lupus-prone NZM2410 and NZB-W/F1 mice and age-matched BALB/c and C57BL/6 control mice. Lupus DCs showed an altered *ex vivo *costimulatory profile, with a significant increase in the expression of CD40, decreased expression of CD80 and CD54, and normal expression of CD86. DCs from young lupus-prone NZM2410 mice, before the development of the disease, expressed normal levels of CD80 and CD86 but already overexpressed CD40. The increase in CD40-positive cells was specific for DCs and involved the subset of myeloid and CD8α^+ ^DCs before disease onset, with a small involvement of plasmacytoid DCs in diseased mice. *In vitro *data from bone marrow-derived DCs and splenic myeloid DCs suggest that the overexpression of CD40 is not due to a primary alteration of CD40 regulation in DCs but rather to an extrinsic stimulus. Our analyses suggest that the defect of CD80 in NZM2410 and NZB-W/F1 mice, which closely resembles the costimulatory defect found in DCs from humans with systemic lupus erythematosus, is linked to the autoimmune disease. The increase in CD40 may instead participate in disease pathogenesis, being present months before any sign of autoimmunity, and its downregulation should be explored as an alternative to treatment with anti-CD40 ligand in lupus.

## Introduction

Dendritic cells (DCs) initiate and maintain immune responses, and evidence suggests that they have an active role in the pathogenesis of lupus: human DCs progressively disappear from the blood of patients with lupus [1], possibly because they are sequestered in the secondary lymphoid organs; they show an abnormal costimulatory profile [2], with a specific defect of expression of CD80 [3,4]; and sera from patients with lupus have been shown to induce differentiation of peripheral blood mononuclear cells into DCs, with a mechanism dependent on type I IFN-α [5], a cytokine produced in large amounts by plasmacytoid DCs (pDCs) [6]. DCs accumulate both in T cell areas [7] and B cell areas [8] in the lymph nodes and spleens of lupus-prone MRL/*lpr *(Fas-deficient) and NZB-W/F1 mice; in the latter strain, the accumulation has been attributed to high levels of Flt-3L [9], which is also increased in patients with lupus [10]. In (SWR × NZB) F_1 _mice, DC accumulation was inhibited by long-term treatment with anti-CD40 ligand (anti-CD40L), suggesting that it might be dependent on CD40-CD40L interaction [11]. Finally, injection of syngeneic DCs accelerates the onset of the disease [12]. The antigen-presenting capability of DCs in lupus-prone mice requires further characterization, because normal [9], defective [13], and excessive [14] antigen-presenting cell functions have all been reported in different murine models of lupus.

Although many studies have focused on the relevance of DCs in lupus-prone mice [9,11,15], the costimulatory phenotype of DCs *ex vivo *has not been thoroughly investigated, especially without the limitations of isolation *in vitro*. A recent report describes the phenotype of DCs in C57BL/6.*Sle*3 mice, a congenic strain of mice in which one of the NZM2410-derived lupus susceptibility loci, *Sle*3, was introgressed onto the normal C57BL/6 (B6) background [14]. These mice have low levels of autoantibodies and lymphocyte alterations but do not develop overt lupus disease with the characteristic lupus nephritis. DCs from B6.*Sle*3 mice showed higher levels of expression of costimulatory molecules, elevated production of cytokines, and increased T cell stimulatory capacity before and after the onset of the disease [14].

In our study we analyzed DCs in the original NZM2410 and NZB-W/F1 mice before and after the onset of overt disease and found different results from those reported in B6.*Sle*3 mice. We used a protocol that maximizes DC yield [16] to analyze the characteristics of the three major DC subsets, namely myeloid, CD8α^+^, and plasmacytoid; and to compare lupus-prone mice with multiple age-matched non-autoimmune mice. We show here that DCs from diseased NZM2410 lupus-prone mice have an altered costimulation pattern, with an abnormal ratio of CD80 to CD86 due to decreased levels of CD80 and normal levels of CD86. Additionally, we detected decreased levels of CD54, and a specific increase in CD40 on lupus DCs. Whereas the alteration in CD80/CD86 ratio is evident only after the onset of the disease, the overexpression of CD40 precedes the onset of lupus and is sustained even during the course of the disease. We therefore propose that the decreased ratio of CD80 to CD86 in lupus DCs may be linked to the full development of the autoimmune disease, whereas the overexpression of CD40 may be important in the pathogenesis of this autoimmune disease.

## Materials and methods

### Mice

NZM2410 (Taconic and Jackson Laboratories), NZB-ZW/F1J, BALB/c, and C57BL/6J (Jackson Laboratories) mice were bred and maintained in accordance with the guidelines of the IACUC and all the experimental procedures were approved by the IACUC of the Children's Hospital of Philadelphia, an AAALAC accredited facility.

### Immunostaining for flow cytometry *ex vivo*

To study DCs in the spleen *ex vivo*, we used a modified protocol from Vremec and colleagues [16]. In brief, we injected excised spleens with a solution of 0.8 mg/ml collagenase and 0.1 mg/ml DNAse, then teased the tissue into small pieces, and incubated it for 30 minutes at 37°C. From this point onward, all procedures were performed on ice to minimize the spontaneous activation of DCs *in vitro*. We pushed spleen fragments through a cell strainer (100 μm) and inhibited collagenase with 5 mM EDTA; then we lysed the red blood cells with Hybrimax (Sigma, St Louis, MO, USA) and counted the cells. We prestained the total population of splenocytes for 10 minutes with rat anti-mouse CD16/CD32 (clone 2.4G2) antibodies to block FcγR, and then stained for 30 minutes with the following monoclonal antibodies from BD Bioscience Pharmingen (San Diego, CA, USA): Allophycocyanin -conjugated hamster anti-mouse CD11c (to recognize DCs) and PerCP-Cy5.5-rat anti-mouse CD19 (to gate out CD11c^low ^B cells). In some experiments, biotinylated anti-F4/80 and NK1.1 monoclonal antibodies, followed by Streptavidin PerCP-Cy5.5 were used to gate out CD11c^low ^macrophages and natural killer cells, respectively. We found that these cells do not contribute significantly to the CD11c^+ ^population.

We studied DC costimulatory molecules with phycoerythrin-conjugated rat anti-mouse CD80, CD86, and CD54 and with fluorescein isothiocyanate-conjugated hamster anti-mouse CD40. To study CD40 expression in DC subsets, we used phycoerythrin-conjugated rat anti-mouse CD8α, CD11b, and B220. In parallel tubes we stained cells with isotype control antibodies to determine non-specific staining. After staining, cells were washed in phosphate-buffered saline, fixed in 1% formaldehyde, and analyzed on a FACSCalibur flow cytometer (BD Biosciences Pharmingen, San Diego, CA, USA). We collected a large number of cells per sample (5 × 10^5 ^cells per tube), allowing us to study small populations among the splenocytes, such as the CD11c^+ ^DCs (about 2 to 4% of the splenocytes) and the smaller subsets: the myeloid DCs (recognized as CD11c^+ ^CD11b^+ ^cells), the pDCs and the CD8α^+ ^DCs (recognized as CD11c^+ ^B220^+ ^cells and CD11c^+ ^CD8α^+ ^cells, respectively). The large number of cells yielded a sufficiently large number of events (more than 2,000 for subsets, and up to 18,000 for the all DC populations) to guarantee reliable results.

### Bone marrow-derived DCs

We generated bone marrow-derived DCs (BM-DCs) as described previously [17]. In brief, we eliminated T cell and B cell contaminants from bone marrow with anti-Thy 1.2 and anti-B220 magnetic beads and MACS columns (Miltenyi Biotech, Bergisch Gladbach, Germany); then seeded bone marrow precursors in 24-well plates at 10^6 ^cells/ml in 10% fetal bovine serum in complete IMDM (Iscove's modified Dulbecco's medium) containing glutamine, 2-mercaptoethanol, and antibiotics, and enriched with 3 ng/ml granulocyte/macrophage colony-stimulating factor (GM-CSF) and 2.5 ng/ml interleukin-4 (BD Bioscience Pharmingen, San Diego, CA, USA). We used BM-DCs at day 6 to 7 of culture.

### Isolation of splenic myeloid DCs

We isolated splenic myeloid DCs as described previously [18]. In brief, we freed the DCs from the extracellular matrix, as described above. We plated the splenocytes on polystyrene Petri dishes to let DCs and macrophages adhere, and washed out the non-adherent cells after 90 minutes. We then incubated the adherent cells overnight in complete medium enriched with 10 ng/ml GM-CSF. On the following day, we collected the floating DCs while leaving the strongly adherent macrophages on the plastic. After harvesting, we stained the isolated splenic DCs as described above.

### Statistical analyses

We analyzed the results with the non-parametric Kruskal-Wallis test and the non-parametric post hoc Mann-Whitney test to evaluate the differences between experimental groups. We considered *p *< 0.05 to be significant.

## Results

### DCs from lupus-prone mice accumulate with age

We studied DCs in spleens of lupus-prone NZM2410 mice in comparison with non-autoimmune age-matched BALB/c and B6 mice. NZM2410 mice, derived by selective inbreeding of the progeny of NZB-W/F1 mice, resemble, both serologically and clinically, the manifestations of human systemic lupus erythematosus (SLE) [19]. Because the parental NZB and NZW mice display some autoimmune features, we chose as controls two non-autoimmune strains of mice, B6 and BALB/c, which in the literature are the most commonly used for comparison with NZM2410 [20,21] and NZB-W/F1 mice [12,22], respectively. Differences shared by these two non-autoimmune mice in comparison with the NZM2410 mice are likely to be lupus specific. We first studied mice with lupus at 6 to 9 months of age (adult mice). The prevalence of the autoimmune disease is about 90% at this age. For our experiments, we selected mice that showed high titers of anti-DNA antibodies in their sera, detected with a specific ELISA (not shown). Additionally, we assessed kidney damage by detecting elevated proteinuria (more than 300 mg/dl) and blood urea nitrogen (more than 26 mg/dl), and an increase in body weight (45 to 50 g) due to fluid retention from kidney failure (not shown). Age-matched BALB/c and B6 mice did not have anti-DNA antibodies and displayed levels of proteinuria in the normal range for mice (30 to 100 mg/dl), normal blood urea nitrogen (less than 26 mg/dl), and stable adult body weight (25 to 30 g) (not shown).

We also investigated DCs from spleens of mice 6 to 8 weeks old (young). At this age, no sign of autoimmunity (anti-DNA antibodies or kidney damage) is evident in the NZM2410 or in the NZB-W/F1 model of lupus.

**Figure 1:**
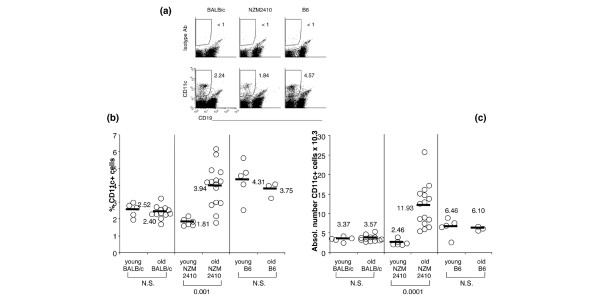
DCs accumulate with age in mice with lupus. **(a) **To study DCs, we analyzed the total population of splenocytes in mice 6 to 8 weeks old (young) and in mice 6 to 9 months old (old) and gated for CD11c^+ ^cells that were negative for the B cell marker CD19. **(b,c) **Percentages (b) and absolute numbers (c) of DCs gated as in (a). Each circle represents the percentage or absolute number of CD11c^+ ^cells in one individual mouse, and the horizontal bars represent the average of each group. Results of the Mann-Whitney test analyses are shown. N.S. indicates *p *> 0.05.

To study DCs *ex vivo *we performed no cell selection, to avoid any loss of DC subsets. We therefore stained the entire population of splenocytes and analyzed a large number of cells by flow cytometry (see details in Methods). Figure 1a shows the gate used to analyze DCs. We found that, after the onset of disease, splenic DCs accumulate with age (Figure 1b,c). Indeed, we found significant differences in percentages and absolute numbers of DCs between young and adult NZM2410 mice. Such accumulation with age is not present in either BALB/c or B6 mice: in these strains, DC percentages and absolute numbers were not significantly different in young and adult mice (Figure 1b,c). These data are consistent with the significant difference in CD11c^+ ^DC numbers between lupus-prone and BALB/c adult mice reported in NZW-BXSB/F1 [9] and in NZB-W/F1 mice (LC, JD, DKS, LF, RC, SG, unpublished data). In addition, we propose the new concept that the distribution of DCs is a variable among different strains (compare B6 with BALB/c) and that DC accumulation needs to be measured by comparing different ages in a single strain. These observations suggest that DC accumulation is common to at least three murine models of lupus [8,9,11] and therefore that it is likely to be important in lupus development.

### DCs from lupus mice express an altered ratio of CD80 to CD86 *ex vivo*

**Figure 2:**
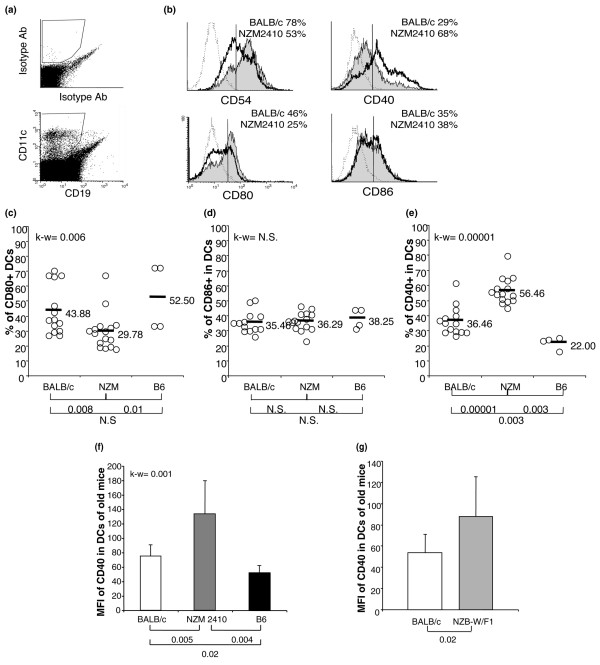
DCs express an altered costimulatory phenotype in lupus diseased mice. **(a) **To study DCs, we analyzed the total population of splenocytes in mice 6 to 9 months old and gated for CD11c^+ ^cells that were negative for the B-cell marker CD19. **(b) **DCs from mice with lupus (thick dark histograms) express lower levels of CD80, normal levels of CD86, and higher levels of CD40 than DCs of non-autoimmune mice (grey filled histograms). The highlighted numbers are the percentages of DCs positive for the indicated markers and are representative of several mice analyzed and shown in (c-e). The vertical line delineates the threshold of positivity, set on the isotype control background less than 1% (dotted histograms). **(c-e) **Positivity for CD80 (c), CD86 (d), and CD40 (e) in DCs gated as in (a). Each circle represents the percentage of positive cells in one individual mouse, and the horizontal bars represent the average of each group. **(f,g) **Averages and standard deviations of CD40 mean fluorescence intensity for expression in DCs from mice 6 to 9 months old of the indicated strains. We analyzed four to nine mice per group in (f) and seven to nine mice per group in (g). Results of the Kruskal-Wallis test analyses are shown in the graphs (k-w) and the post hoc Mann-Whitney test analyses are shown below the graphs. N.S. indicates *p *> 0.05.

The analysis of the costimulatory molecules expressed by DCs *ex vivo *(Figure 2a,b) showed that NZM2410 mice 6 to 9 months old (adult) had a lower percentage of DCs expressing CD80, whereas the positivity for CD86 was similar to that of the non-autoimmune mice (Figure 2c–e). Moreover, the comparable levels of CD80 and CD86 in BALB/c and B6 DCs confirmed the feasibility of using these two strains of mice for comparison with the lupus-prone strain. As was expected, we found a similar profile (normal CD86 and low CD80) in age-matched NZB-W/F1 mice (not shown). Furthermore, in both NZM2410 and NZB-W/F1 mice, we found a decreased expression of the adhesion molecule CD54 on lupus DCs in comparison with non-autoimmune age-matched BALB/c DCs (Figure 2b and not shown).

A defect in the expression of the costimulatory molecule CD80 and the adhesion molecule CD54, in the presence of normal levels of the costimulatory molecule CD86, suggests a pathologic state of activation of DCs arising during the disease.

### Increase in CD40-positive DCs in diseased mice *ex vivo*

We also found that CD40 expression was highly significantly upregulated in DCs from spleens of diseased NZM2410 mice in comparison with age-matched non-autoimmune mice (Figure 2b,e). The increase in the percentage of CD40-positive DCs was accompanied by an increase in CD40 mean fluorescence intensity (MFI) (Figure 2f). The levels of CD40 positivity in DCs of the two non-autoimmune strains were significantly different, but they were both much lower than in lupus DCs (Figure 2e,f). We found the same constitutive overexpression of CD40 in NZB-W/F1 DCs (Figure 2g). The increase in CD40 MFI was not accompanied by an increase in the maximal levels of CD40 expression (Figure 2b). These data suggest an increased number of activated DCs rather than an increased capacity of lupus DCs to express CD40 once activated.

#### The altered CD80/CD86 ratio does not precede the onset of lupus disease

**Figure 3:**
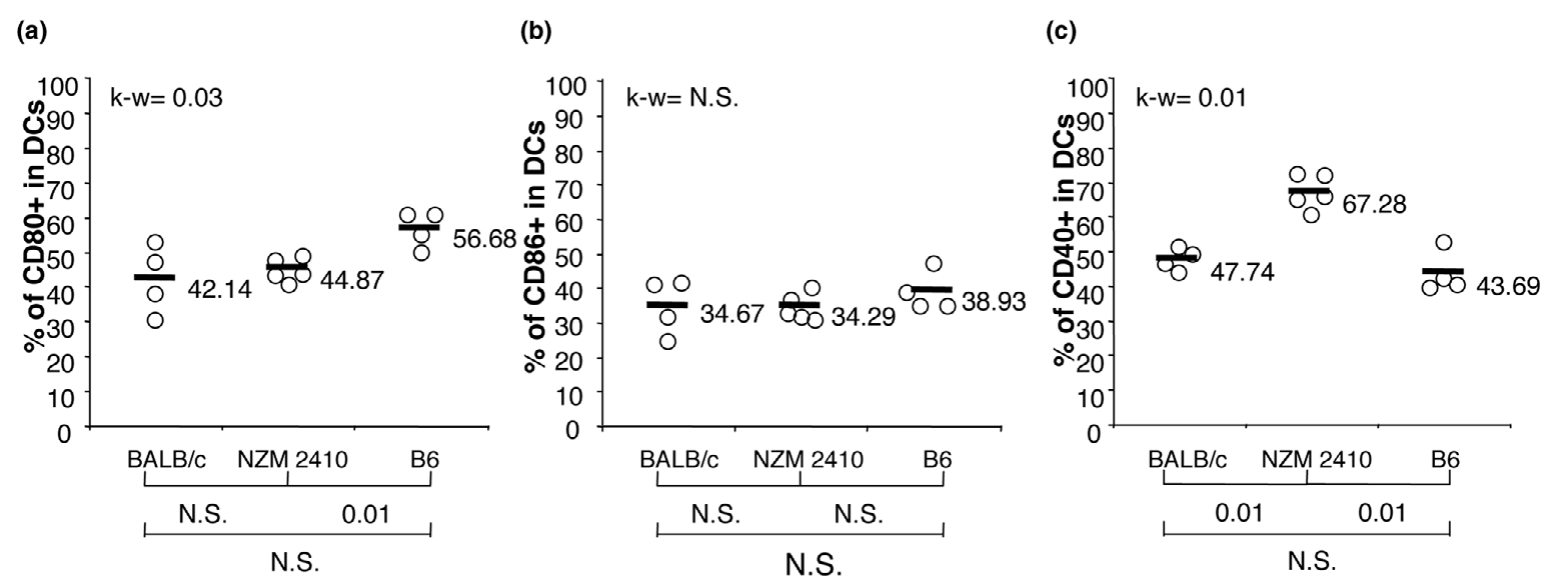
Increase in CD40-positive DCs pre-dates lupus onset of disease, whereas CD80/CD86 balance is normal. Percentages of cells positive for CD80 **(a)**, CD86 **(b)**, and CD40 **(c) **in DCs from spleens of young (6 to 8 weeks old) mice. We analyzed DCs as described in Figure 2. Results of the Kruskal-Wallis test analyses are shown in the graphs (k-w) and the post hoc Mann-Whitney test analyses are shown below the graphs. N.S. indicates *p *> 0.05.

We asked whether alterations of the activation markers in lupus DCs were secondary to the inflammatory process that characterizes the autoimmune disease, or whether they were instead primary events and possibly involved in lupus pathogenesis. We therefore investigated the costimulatory phenotype of DCs from spleens of mice 6 to 8 weeks old (young). At this age, no sign of autoimmunity (anti-DNA antibodies or kidney damage) is evident in the NZM2410 or in the NZB-W/F1 model of lupus. We found that DCs from lupus-prone mice expressed levels of CD80 and CD86 comparable to those ofBALB/c and B6 DCs, in terms of both percentage of positive cells (Figure 3a,b) and MFI (not shown). In the two control strains, the expression of costimulatory molecules CD80 and CD86 shows very consistent percentages in young and adult mice (CD80: BALB/c 42.12% (young) versus 43.88% (adult); B6 56.68% (young) versus 52.50% (adult); CD86: BALB/c 34.67% (young) versus 35.46% (adult); B6 38.93% (young) versus 38.25% (adult)). We found the same consistency in NZM2410 DCs for CD86 (34.29% (young) versus 36.29% (adult)), but not for CD80 (44.87% (young) versus 29.78% (adult)) (compare Figure 2c,d with Figure 3a). Because these data were collected from a large number of mice, especially in the adult populations, and in separate experiments over a period of months, such consistency strengthens the significance of the altered expression ratio of CD80 to CD86 observed in adult mice with lupus.

### The increase in CD40-positive DCs precedes the onset of lupus disease

We also investigated CD40 expression in the same group of young mice. We found that young NZM2410 mice have a higher percentage of CD40-positive DCs in their spleens than either BALB/c or B6 mice. The two control strains had very similar levels of CD40-positive DCs (Figure 3c), both significantly lower than in NZM2410 mice. The percentages of CD40-positive DCs decreased with age in the three strains, with a more pronounced effect in B6 mice (compare Figure 2e with Figure 3c). Although the functional significance of these age-related differences is worth further investigation, our results show that DCs from lupus-prone mice express abnormal and long-lasting levels of this fundamental marker.

In summary, we found that DCs from lupus-prone mice show a pathologic state of activation in comparison with age-matched control mice. Our analysis suggests that the decreased CD80/CD86 ratio may be linked to the full development of the autoimmune disease, whereas the overexpression of CD40 pre-dates the occurrence of overt autoimmunity and may therefore be involved in its pathogenesis.

### The increase in CD40 positivity is specific to DCs

**Figure 4:**
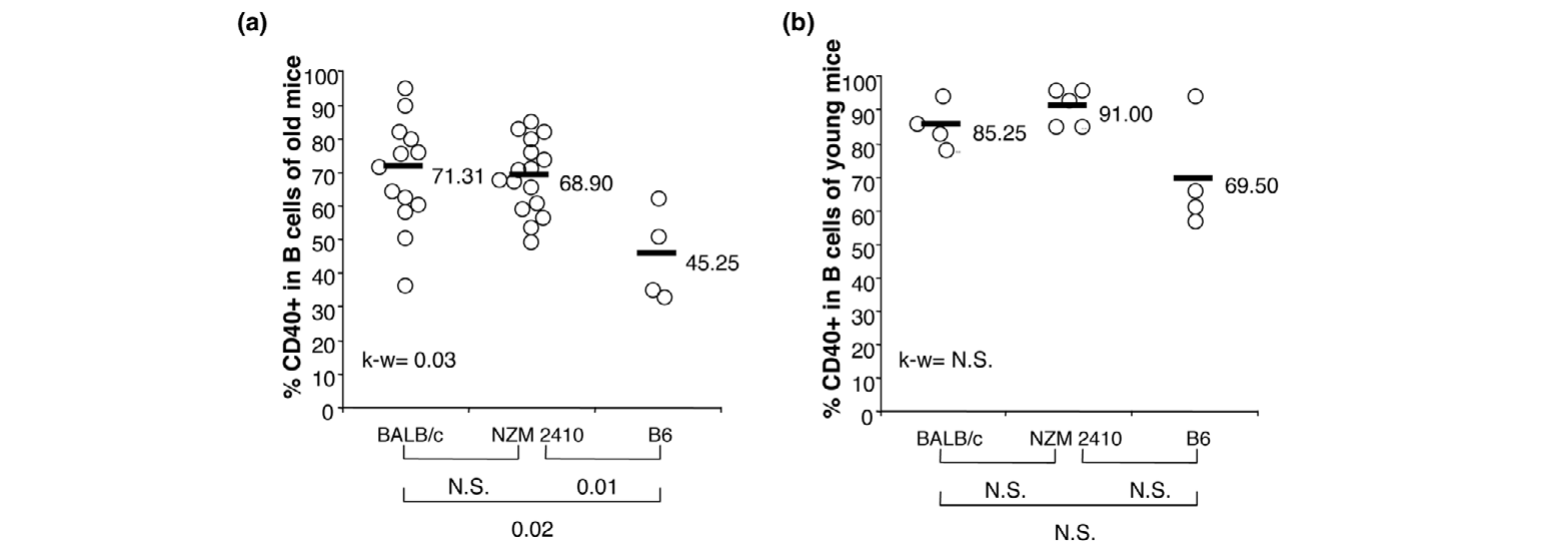
B cells from lupus-prone mice express normal levels of CD40. Percentages of B cells positive for CD40 from mice 6 to 9 months old **(a) **and 6 to 8 weeks old **(b) **are shown. B cells were gated as B220/CD19-positive and CD11c-negative cells. Results of the Kruskal-Wallis test analyses are shown in the graphs (k-w) and the post hoc Mann-Whitney test analyses are shown below the graphs. N.S. indicates *p *> 0.05.

To determine whether the overexpression of CD40 was a DC-specific phenomenon, we analyzed the expression of CD40 in B cells, the other important population of immune cells that normally express this marker. As expected, a large percentage of B cells constitutively expressed high levels of CD40. We found that NZM2410 mice and age-matched BALB/c mice had similar percentages of CD40-positive B cells, both in adult animals (Figure 4a) and in young animals (Figure 4b), whereas B6 B cells showed overall lower levels of expression, especially in adult mice.

Because the percentage of CD40-positive B cells in NZM2410 mice was significantly higher only in comparison with B6 but not with BALB/c mice, and this single difference was present only after onset of the disease, we propose that the overexpression of CD40 in lupus-prone mice is DC specific.

### Analysis of CD40 expression in DC subsets

**Figure 5:**
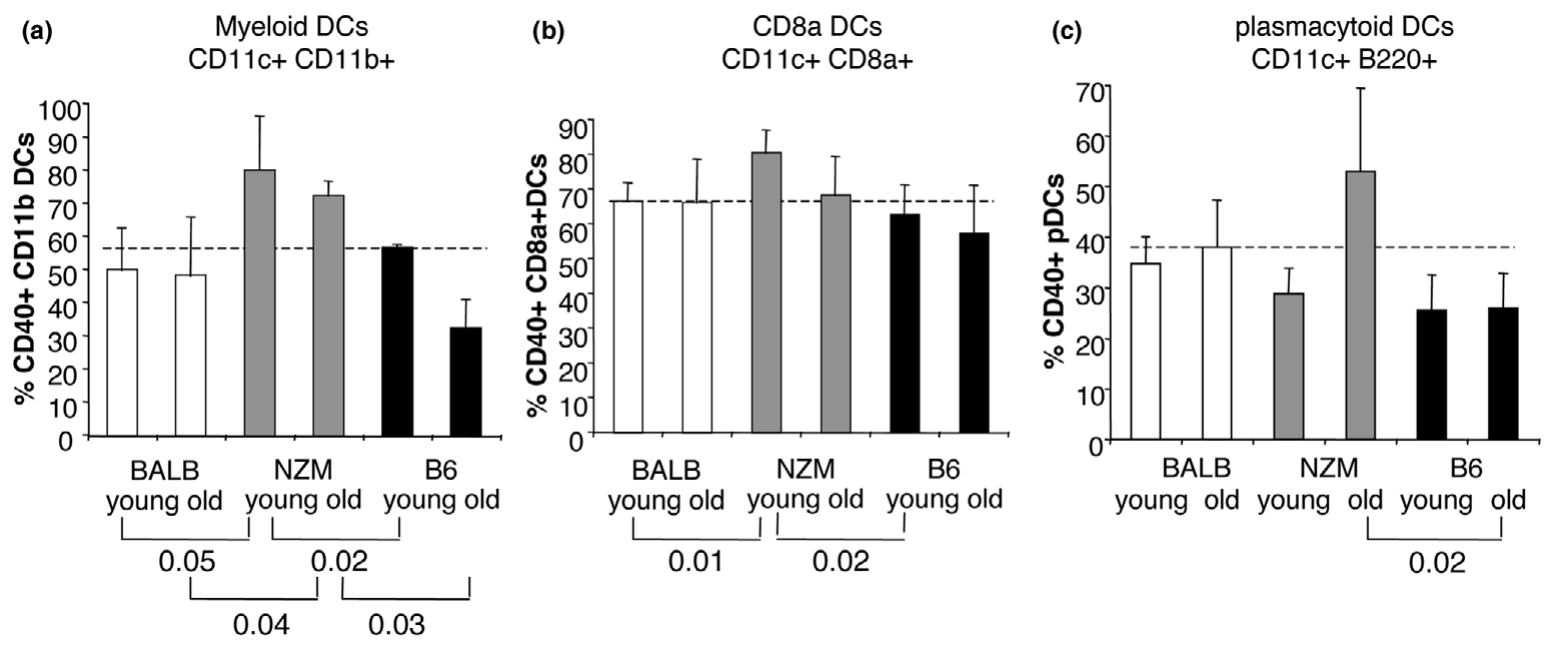
CD40 expression in three major DC subsets. Percentages and standard deviations of CD40-positive cells in myeloid **(a)**, CD8α^+ ^**(b)**, and plasmacytoid **(c) **DC subsets from young (6 to 8 weeks old) and old (6 to 9 months old) mice were calculated from four to nine mice per group. Only results by the Mann-Whitney test analyses with *p *< 0.05 are indicated.

DCs can be subdivided into subsets, characterized by specific surface markers and functions [23]. We therefore asked which DC subset is responsible for the increase in CD40-positive DCs in lupus-prone mice. In young NZM2410 mice, we observed overexpression of CD40 largely associated with myeloid DCs. This subset continued to overexpress CD40 after the onset of the disease (Figure 5a). CD8α^+ ^DCs constitutively expressed high levels of CD40 in all three strains (Figure 5b). Nevertheless, we observed a slightly but significantly higher percentage of CD40-positive cells in CD8α^+ ^DCs from lupus-prone mice than in non-autoimmune mice at a young age. Such overexpression disappeared after lupus onset (Figure 5b). The pDCs, which normally present a more immature phenotype than other subsets, expressed low levels of CD40 in all three strains, with normal percentages of CD40 positivity in young lupus-prone mice (Figure 5c) and increased percentages after lupus onset, although the increase was not statistically significant (Figure 5c). Because we found greater variability in the CD40 expression of pDCs from old mice with lupus than from the other groups of mice, we take into account that technical reasons may have hampered the statistical significance in this comparison (Kruskal-Wallis *p *= 0.018, Mann-Whitney NZM versus BALB/c = 0.064, NZM versus B6 = 0.023). We therefore do not completely exclude the involvement of pDCs in the increased DC expression of CD40 after the onset of the disease.

This thorough analysis suggests that the increase in CD40-positive DCs is due mainly to the myeloid DCs and to a smaller extent, before the onset of the disease, to the CD8α^+ ^DCs, and after disease onset, to pDCs.

### The overexpression of CD40 is not constitutive in resting DCs

The increase in the percentage of CD40-positive DCs that we observed in NZM2410 and in NZB-W/F1 mice could be due to a pro-inflammatory environment present in lupus-prone mice even months before the onset of the disease, or to a primary alteration in the state of activation of DCs.

**Figure 6:**
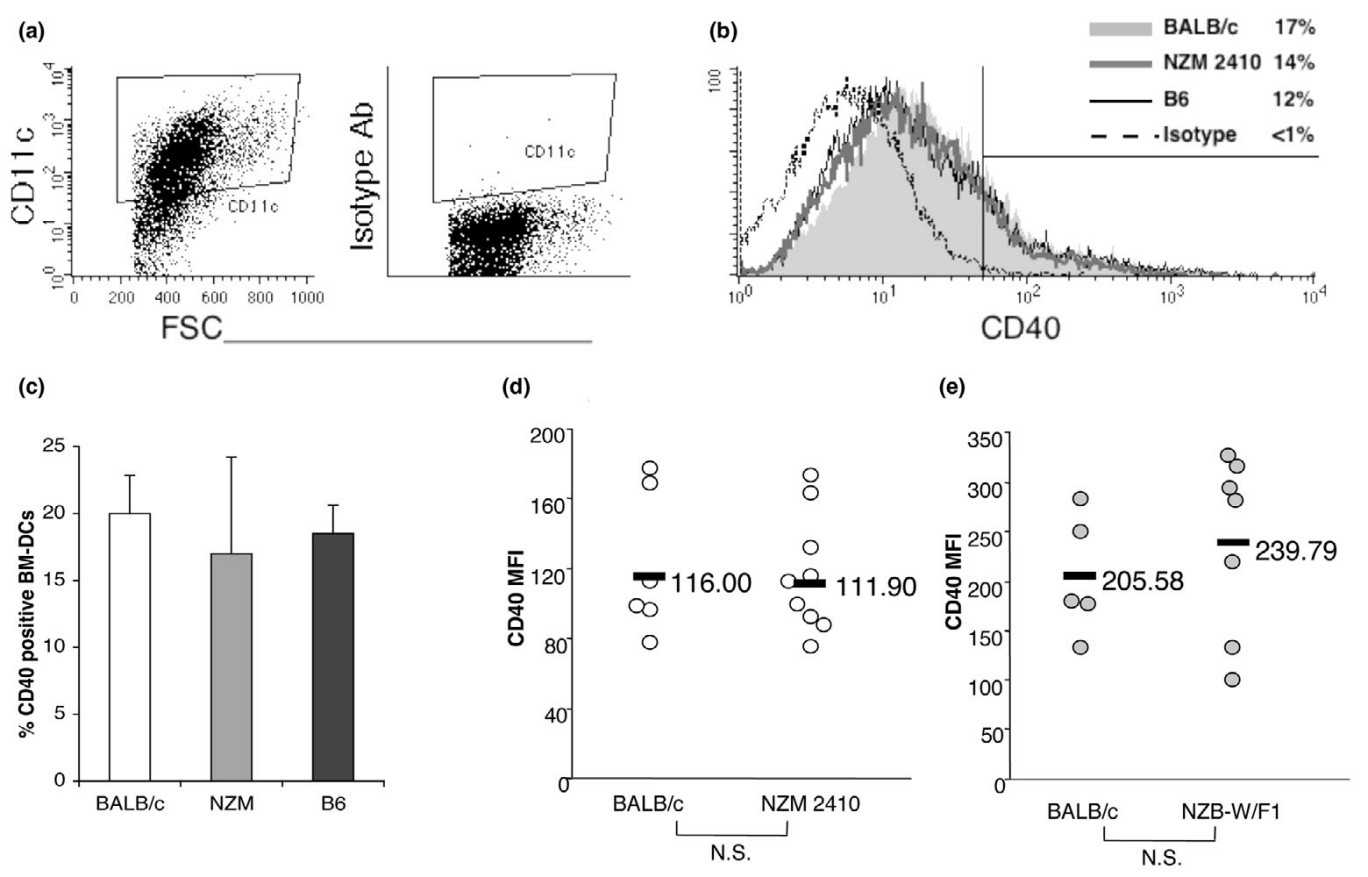
Regulation of CD40 expression is not intrinsically altered in lupus DCs. We grew bone marrow-derived dendritic cells (BM-DCs) from NZM2410, BALB/c, and B6 mice 6 to 9 months old and stained them at day 6 to 7 of culture. Gating of CD11c^+ ^cells **(a) **and CD40 expression **(b) **in resting BM-DCs from NZM2410 (thick dark grey line), BALB/c (light grey area), and B6 (thin black line) mice. **(c) **Percentages of CD40-positive BM-DCs, gated as in (a) and considered CD40-positive using the threshold shown in (b) from three experiments, conducted with three independent BM-DCs cultures. Error bars show SD. **(d,e) **Mean fluorescence intensity (MFI) of CD40 expression in splenic myeloid DCs isolated and cultured overnight *in vitro *from NZM2410 (**d**) and NZB-W/F1 (**e**) mice 6 to 9 months old.

To discriminate intrinsic DC abnormalities from those induced by environmental factors, we analyzed CD40 expression in NZM2410, BALB/c, and B6 BM-DCs, grown in culture with a protocol that generates resting CD11c^+ ^CD11b^+ ^myeloid-like DCs [17]. In this artificial environment, DCs are influenced only by themselves and by the cytokines that are provided to them in culture (Figure 6a). We found that lupus BM-DCs expressed levels of CD40 comparable to those of BALB/c and B6 BM-DCs in terms of both percentage of positivity (Figure 6b,c) and MFI (Figure 6b). We found the same results in BM-DCs grown from both old (Figure 6a–c) and young mice (data not shown).

These results suggest that lupus DCs do not have a primary alteration in their expression of CD40, but that the overexpression observed *ex vivo* may be the result of a chronic exposure or altered response to activators *in vivo*.

### Lupus DCs have a normal capacity to upregulate CD40 expression after activation

To determine whether lupus DCs have a normal capacity to upregulate CD40 expression after activation, we used the traditional protocol by Inaba and colleagues to isolate splenic DCs [18]. This protocol gives rise to a pure (more than 90%; data not shown) population of cells double positive for CD11b and CD11c and negative for CD8α or B220, therefore resembling myeloid DCs. Moreover, DCs are highly activated by the procedure, with more than 80% of DCs expressing high levels of costimulatory molecules (not shown). Although the stimulus that induces DC activation is unclear, this protocol allowed us to analyze the intrinsic capacity of DCs to upregulate CD40 [18,24]. We found that lupus DCs from both NZM2410 mice (Figure 6d) and NZB-W/F1 mice (Figure 6e) upregulated CD40 to the same extent as the non-autoimmune DCs, therefore suggesting that the regulation of CD40 expression is not intrinsically altered.

## Discussion

We have examined the *ex vivo *costimulatory phenotype of DCs from NZM2410 and NZB-W/F1 lupus-prone mice, compared with DCs from age-matched non-autoimmune mice. We found that in lupus-prone mice, after the onset of the disease, DCs expressed an abnormal state of activation with decreased levels of CD80 and CD54, normal levels of CD86, and a specific increase in cells expressing CD40. Importantly, the CD40 increase was already present months before the onset of the disease. The overexpression of CD40 may be important in SLE development because CD40 triggering could be responsible for the inappropriate stimulation of DCs to live longer, produce an excess of pro-inflammatory cytokines, and deliver abnormal activation signals to autoreactive T and B cells.

In the pre-disease stage, the increase in CD40 positivity was mostly present in myeloid DCs and, to a smaller extent, in CD8α^+ ^DCs. Both DC subsets have been proposed to induce peripheral tolerance [25] unless they receive an activation signal, such as CD40 triggering, that induces the production by DCs of large amounts of pro-inflammatory cytokines and stimulates T cells [26]. Our results therefore suggest that in young lupus-prone mice, months before the appearance of any autoantibodies, DCs are prone to escape from a tolerogenic status.

The small pDC subset has been proposed to be important in lupus pathogenesis [27] because pDCs can produce large amounts of type I IFN, which is considered altered in this disease [5]. CD40 triggering was one of the first stimuli shown to induce the production of type I IFN by pDCs [28]. Although the increase in CD40 expression that we found in pDCs, after the onset of lupus, did not reach statistical significance with non-parametric tests, we cannot exclude CD40-CD40L interactions as a mechanism by which pDCs are induced to overproduce type I IFN and sustain autoimmunity.

We have found that resting lupus BM-DCs, differentiated in the artificial environment of culture *in vitro*, did not express increased levels of CD40. Furthermore, splenic DCs from mice with lupus showed a normal upregulation of CD40 expression on activation *in vitro*. These results suggest that lupus DCs do not have a primary and constitutive alteration in their regulation of CD40, but they may be induced to overexpress CD40 by a chronic stimulus.

The decreased CD80/CD86 ratio in DCs from NZM2410 mice after the onset of the disease resembles the defective costimulatory profile found in DCs from patients with SLE [2,4] and validates the use of NZM2410 and NZB-W/F1 strains as murine models for the study of human SLE. CD80 and CD86 are ligands of CD28/CTLA4 and may have different functions [29]: although both CD80 and CD86 activate effector T cells, CD80 seems to be especially important for the stimulation of T regulatory cells and, therefore, the inhibition of the immune response. Indeed, DNA immunization with plasmids encoding CD80 induces weaker responses than with plasmids encoding CD86 [30]. In diabetic mice and in lupus-prone MRL/*lpr *mice, the blockade of CD80 worsens the severity of both diseases, whereas blockade of CD86 prevents diabetes and has mild effects on lupus [31,32]. These data suggest the fascinating hypothesis that the unbalanced expression of CD80 and CD86 in lupus DCs may impair the capacity of DCs to engage regulatory T cells, which would lead to the inappropriate stimulation of autoreactive B cells and the maintenance of the autoimmune disorder.

Recently, Zhu and colleagues [14] reported that DCs from NZM2410 single-locus derivative B6.*Sle3 *mice are hyperactivated and hyperstimulate T cells. Some of our results agree with their data, but some differ. Indeed, they found, as we did, that DCs from the spleens of mice 9 to 12 months old hyperstimulate T cells (data not shown). However, they also demonstrated increased expression of CD40, CD54, CD80, and CD86, with a normal CD80/CD86 ratio, whereas we found an increased expression of CD40 with decreased levels of CD80 and CD54. Although we cannot exclude technical reasons to explain the dissimilarities, we think that genetic and disease differences between the two strains of mice can account for the discrepancies. Indeed, NZM2410 mice develop an overt form of lupus, with the full repertoire of autoimmune features and severe glomerulonephritis, whereas B6.*Sle3 *mice display some autoimmune features but do not develop glomerulonephritis. We therefore propose that NZM2410 mice and their single-locus derivative B6.*Sle3 *mice have a genetic abnormality, still to be determined and not intrinsic to DCs, that induces upregulation of CD40 in DCs *in vivo *before and after the onset of the disease. In NZM2410 mice, the involvement of other immune abnormalities would lead to a decreased CD80/CD86 ratio on DCs, and ultimately to severe overt disease, hypothetically because of impaired activation of regulatory T cells.

CD40-CD40L interaction has been found to be involved in the pathogenesis of lupus in four murine models [33-37], and ectopic expression of CD40L on B cells induces a lupus-like autoimmune disease [38]. In patients with SLE, T cells overexpress CD40L for extended periods [39,40], and activated B cells express CD40L ectopically [41]. Analysis of the literature suggests that the overexpression of CD40 and CD40L may be independent phenomena and may both be required for full lupus development.

Long-term administration of anti-CD40L antibody prevents the production of high-titer autoantibodies and disease onset in mice [34,36,42]. In (SWRxNZB)F1 mice, it also prevented DC accumulation, suggesting that it may be CD40-CD40L dependent [11]. Our finding of the increase in CD40-expressing DCs in lupus-prone mice before the onset of the disease suggests that CD40 on DCs may have a role in the pathogenesis of this autoimmune disease. We therefore propose that the marked effects of the blockade of CD40L in patients and murine model of lupus are also due to the inhibition of CD40 triggering on DCs. Because anti-CD40L blockade has raised safety concerns in patients with SLE [43], we propose that the inhibition of the expression and function of CD40 in DCs may be an alternative therapeutic strategy.

## Conclusion

Our results suggest that the dendritic cells, pivotal links between the innate and the adaptive immune system, play an important role in the pathogenesis of the autoimmune disease lupus and should be considered as a therapeutic target in SLE through a specific intervention on the expression of costimulatory molecules.

## Abbreviations

B6 = C57BL/6; BM-DCs = bone marrow-derived dendritic cells; CD40L = CD40 ligand; DCs = dendritic cells; ELISA = enzyme-linked immunosorbent assay; IFN = interferon; MFI = mean fluorescence intensity; pDCs = plasmacytoid dendritic cells; SLE = systemic lupus erythematosus.

## Competing interests

The authors declare that they have no competing interests.

## Authors' contributions

LC performed many of the immunostainings to characterize DC phenotype, analyzed the data, and drafted the manuscript. JD performed many of the immunostainings to characterize DC phenotype and analyzed the data. DKS monitored the disease in mice with lupus. LF performed the ELISA anti-DNA and analyzed the data. RC participated in the design of the study and helped to draft the manuscript. SG conceived of the study, and participated in its design and coordination, and helped to draft the manuscript. All authors read and approved the final manuscript.

## Figures and Tables

**Table 1:** Lupus dendritic cells show a decreased CD80/CD86 ratio *ex vivo*

Mouse strain	CD80/CD86	*p*
BALB/c	1.23 ± 0.39	
NZM	0.79 ± 0.34	0.006
C57BL/6	1.32 ± 0.35	

We calculated CD80/CD86 ratios, dividing the percentage of dendritic cells positive for CD80 by the percentage of dendritic cells positive for CD86 in each mouse (*ex vivo*) analyzed. Ratios are shown as means ± SD, and the Kruskal-Wallis *p *value for these ratios is shown.
